# Spirometry services in England post-pandemic and the potential role of AI support software: a qualitative study of challenges and opportunities

**DOI:** 10.3399/BJGP.2022.0608

**Published:** 2023-10-31

**Authors:** Gillian Doe, Stephanie JC Taylor, Marko Topalovic, Richard Russell, Rachael A Evans, Julie Maes, Karolien Van Orshovon, Anthony Sunjaya, David Scott, A Toby Prevost, Ethaar El-Emir, Jennifer Harvey, Nicholas S Hopkinson, Samantha S Kon, Suhani Patel, Ian Jarrold, Nanette Spain, William D-C Man, Ann Hutchinson

**Affiliations:** Department of Respiratory Science, University of Leicester, Leicester, UK.; Wolfson Institute of Population Health, Queen Mary University of London, London, UK.; ArtiQ, Leuven, Belgium.; Nuffield Department of Medicine, University of Oxford, Oxford, UK.; Department of Respiratory Science, University of Leicester, Leicester, UK.; ArtiQ, Leuven, Belgium.; ArtiQ, Leuven, Belgium.; George Institute for Global Health, UNSW Sydney, Australia; George Institute for Global Health, Imperial College London, London; Harefield Respiratory Research Group, Guy’s and St Thomas’ NHS Foundation Trust, London, UK.; Southampton Health Technology Assessments Centre, University of Southampton, Southampton, UK; Diabetes Research Centre, University of Leicester, Leicester, UK.; Nightingale-Saunders Clinical Trials and Epidemiology Unit, Cicely Saunders Institute of Palliative Care, Policy and Rehabilitation, King’s College London, London, UK.; Harefield Respiratory Research Group, Guy’s and St Thomas’ NHS Foundation Trust, London; Department of Respiratory Medicine, Hillingdon Hospitals NHS Foundation Trust, London, UK.; Harefield Respiratory Research Group, Guy’s and St Thomas’ NHS Foundation Trust, London; Department of Respiratory Medicine, Hillingdon Hospitals NHS Foundation Trust, London, UK.; National Heart & Lung Institute, Imperial College London, London, UK.; Harefield Respiratory Research Group, Guy’s and St Thomas’ NHS Foundation Trust, London; Department of Respiratory Medicine, Hillingdon Hospitals NHS Foundation Trust, London, UK.; Harefield Respiratory Research Group, Guy’s and St Thomas’ NHS Foundation Trust, London; National Heart & Lung Institute, Imperial College London, London, UK.; Asthma + Lung UK, London, UK.; Harefield Respiratory Research Group, Guy’s and St Thomas’ NHS Foundation Trust, London; National Heart & Lung Institute, Imperial College London, London; Faculty of Life Sciences and Medicine, King’s College London, London, UK.; Harefield Respiratory Research Group, Guy’s and St Thomas’ NHS Foundation Trust, London; National Heart & Lung Institute, Imperial College London, London; Faculty of Life Sciences and Medicine, King’s College London, London, UK.; Wolfson Palliative Care Research Centre, Hull York Medical School, University of Hull, Hull, UK.

**Keywords:** artificial intelligence, chronic obstructive pulmonary disease, primary care, qualitative research, spirometry, trust

## Abstract

**Background:**

Spirometry services to diagnose and monitor lung disease in primary care were identified as a priority in the NHS Long Term Plan, and are restarting post-COVID-19 pandemic in England; however, evidence regarding best practice is limited.

**Aim:**

To explore perspectives on spirometry provision in primary care, and the potential for artificial intelligence (AI) decision support software to aid quality and interpretation.

**Design and setting:**

Semi-structured interviews with stakeholders in spirometry services across England.

**Method:**

Participants were recruited by snowball sampling. Interviews explored the pre- pandemic delivery of spirometry, restarting of services, and perceptions of the role of AI. Transcripts were analysed thematically.

**Results:**

In total, 28 participants (mean years’ clinical experience = 21.6 [standard deviation 9.4, range 3–40]) were interviewed between April and June 2022. Participants included clinicians (*n* = 25) and commissioners (*n* = 3); eight held regional and/or national respiratory network advisory roles. Four themes were identified: 1) historical challenges in provision of spirometry services; 2) inequity in post- pandemic spirometry provision and challenges to restarting spirometry in primary care; 3) future delivery closer to patients’ homes by appropriately trained staff; and 4) the potential for AI to have supportive roles in spirometry.

**Conclusion:**

Stakeholders highlighted historic challenges and the damaging effects of the pandemic contributing to inequity in provision of spirometry, which must be addressed. Overall, stakeholders were positive about the potential of AI to support clinicians in quality assessment and interpretation of spirometry. However, it was evident that validation of the software must be sufficiently robust for clinicians and healthcare commissioners to have trust in the process.

## INTRODUCTION

Spirometry is a lung function test routinely used to diagnose and monitor chronic lung diseases,^1^ particularly for chronic obstructive pulmonary disease (COPD) and asthma.^2^^,^^3^ In England, spirometry has historically been delivered in GP practices in primary care. However, current spirometry provision is suboptimal: only 13.4% of spirometry performed in primary care meets international criteria,^4^ and 40% of traces fail to meet at least one quality criterion.^5^ There are low levels of confidence in identifying technical errors or interpreting spirometry,^6^ and a low level of agreement on interpretation between primary care and specialist respiratory physicians.^7^

The NHS Long Term Plan^8^ has prioritised improving the quality and provision of spirometry through targeted investment in primary care networks (PCNs). This includes training staff to perform and interpret quality-assured spirometry, and mandating suitable accreditation by the Association for Respiratory Technology and Physiology (ARTP).^9^^,^^10^ Accreditation may be for spirometry test performance only, or test performance and technical interpretation of results.

**Table 1:** How this fits in

Good-quality spirometry to diagnose and monitor lung disease is a priority identified in the NHS Long Term Plan. This study aims to understand perspectives of key stakeholders in spirometry services, in restarting spirometry in primary care post- pandemic, and the potential for artificial intelligence (AI) decision support software. The data highlight the historical challenges to spirometry provision, funding, quality, and interpretation. To improve equitable access to spirometry in primary care, services must be accessible for patients and be delivered by appropriately trained staff. Opportunities arise to reconsider pathway design with potential for AI to support workforce capacity, quality assurance, and interpretation.

The provision of spirometry was negatively impacted by the COVID-19 pandemic,^11^ with infection prevention concerns leading to cessation of spirometry in primary care.^12^^,^^13^ Services are resuming, with national guidance provided to aid the process,^14^ but the pre-pandemic model of delivery may not be reinstated. Coupled with the introduction of integrated care systems,^15^ PCNs, and diagnostic hub models, many services are looking at spirometry pathway redesign.^8^

The interruption to spirometry services during the pandemic, along with reduced help-seeking by patients,^16^ has probably led to an increase in people living with undiagnosed and untreated/mismanaged lung problems.^17^ There are existing health inequities associated with lung disease,^18^ now further exacerbated by inequitable access to diagnostic tests and extensive waiting lists.^19^

Performing quality spirometry is a recognised challenge in primary care, and there is evidence for the use of artificial intelligence (AI) decision support software in improving the quality of both test conduct and interpretation.^20^ Comparison of expert interpretation of spirometry with AI software demonstrated wide variation between expert clinicians in technical interpretation and a correct diagnosis rate of 44%, compared with 82% by the AI software.^20^ This work was conducted among respiratory specialists in a hospital setting, and it is possible that AI could also support GPs who are less experienced in spirometry.

The aim of this study was to understand current provision of spirometry services in primary care and priorities for future delivery, as well as stakeholder views on the potential for AI in spirometry pathways. This qualitative work forms the first stage of an evaluation of the use of an AI decision support software (ArtiQ.Spiro, ArtiQ nv, Leuven, Belgium) in the primary care respiratory diagnostic pathway (https://www.artiq.eu).^21^

## METHOD

This research is reported in line with the Standards for Reporting Qualitative Research.^22^

### Participants

Semi-structured interviews were conducted with key stakeholders involved in commissioning, service design, and the implementation/delivery of spirometry services across England. A snowball sampling strategy was used, starting with existing contacts working in national and local roles within spirometry services.^23^ Participants included national and regional stakeholders with a wide geographical spread. Participants were eligible if involved in spirometry services and able to provide written informed consent.

### Interviews

The authors explored perspectives of key stakeholders on local service provision both pre-pandemic and post-pandemic, priorities for future provision, and views on the potential role of AI decision support software in delivery and interpretation of spirometry. The interview guide (see Supplementary Box S1) was developed with the wider team, drawing on current spirometry guidance and discussion with national respiratory groups. It was developed iteratively throughout the study, based on issues raised in earlier interviews that needed further exploration. Asthma + Lung UK and patient and public involvement representatives were part of the wider study team contributing to the study design.

Interviews were conducted via telephone or online, recorded, and transcribed verbatim. The interviewer had experience of working in primary care, including spirometry delivery and interpretation, and was a trained qualitative researcher. Reflexivity of the interviewer is key in qualitative research and she discussed with the wider team how previous experience influenced her approach to the interviews and interpretation of the data. Interviews were continued until the interview guide was no longer evolving and data were of sufficient depth and complexity around the topic for analysis and identification of themes.^24^^,^^25^

### Analysis

The interview data were analysed using thematic analysis^26^ supported by NVivo (version 12) software for data management. Thematic analysis involves familiarisation with data, generating initial codes, searching for themes, reviewing and naming themes, and producing the report. Initial coding was carried out independently by the interviewer to develop an inductive coding framework.^27^ A sample of transcripts was also coded by two more researchers in the team who had not seen the previous coding, to establish if any new codes were relevant. The wider research team discussed and reviewed the codes and patterns of shared meaning across the transcripts to collaboratively generate themes, using quotes from the transcripts to check data interpretation.

## RESULTS

In total, 28 participants (mean years’ clinical experience = 21.6 [standard deviation 9.4, range 3–40]), of whom 14 were female, five were Asian/Asian British, and 23 were White British, were interviewed between April and June 2022. Participants included clinicians^24^ and commissioners,^3^ and eight held national and/or regional roles influencing policy in this area. Clinician roles from primary and secondary care included seven GPs, six respiratory nurse specialists, eight respiratory consultants, one healthcare assistant, and three respiratory physiologists. Secondary care professionals were included if they also had local or national advisory roles relevant to primary care spirometry pathways. The geographical spread across England included the North West, North East, Midlands, South West, and London.

Four themes were identified and quotes are used below to illustrate each theme.

### Historical challenges in provision of spirometry services

There was wide acknowledgement that level of services varied hugely pre- pandemic:

*‘Pre-pandemic spirometry was being done very widely in primary care, the quality often very variable both in terms of performance and interpretation.’*

(Participant [P]4, consultant)

Participants described many historical challenges to delivering spirometry services in primary care; mode of funding, staff competency, and test quality were important factors.

#### Funding

The inconsistency in mode of funding for spirometry appears to be a longstanding problem, exacerbated by the discrepancy compared with payment in secondary care:

*‘Practices have never been funded to do spirometry* [directly]*, so they’ve all done it because they thought it was a good thing to do. But when they’ve been under pressure … a lot of practices said we’re not funded to do this … I think the hospitals had a system of claiming it as an outpatient appointment, so worth about £160; whereas, the practices with QOF* [diagnosis of COPD confirmed by quality-assured spirometry]*, if they managed to get everybody through it was worth about £7* [per patient on the register]*.’*

(P20, GP)

There was some explanation provided that the lack of direct funding for spirometry was a result of the variable quality, with hope that the NHS Long Term Plan would address this:

*‘Spirometry has never been in the GP contract … primarily because of concerns over quality of spirometry. So what was intended with the Long Term Plan with the diagnostics was that it would become the responsibility of each primary care network to have a spirometry service … ’*

(P27, consultant)

#### Competency to perform spirometry

Accreditation by the ARTP was encouraged but not mandated, and therefore skills and competency were varied, with some suggestion that the accreditation process was difficult to manage in terms of time and motivation. The challenges around accreditation remain a problem in practice:

*‘They* [ARTP] *still haven’t made it mandatory for people to be certified to do spirometry. We did notice anecdotally that a lot of people were doing spirometry and interpreting spirometry without any form of certification.’*

(P7, GP)

*‘The ARTP* [accreditation] *bar is thought to be quite high, and the portfolio and other aspects of that are difficult … ’*

(P28, consultant)

#### Quality of spirometry testing

Many participants described poor-quality assurance in performance and interpretation of spirometry, with reference to lack of awareness of the standards required:

*‘But there wasn’t really any quality assurance and there was quite wide variation in the quality of the spirometry. That was something we were discussing before the pandemic.’*

(P9, consultant)

*‘And by the end of it* [spirometry training] *they say my God, I may have been doing it for twenty years, but I’ve been doing it wrong for twenty years*.*’*

(P23, physiologist)

### Inequity in post-pandemic spirometry provision and challenges to restarting spirometry in primary care

The pandemic halted spirometry in primary care, and, although some services have restarted, participants described several barriers, including infection prevention guidelines and workforce capacity. The variety in current service provision across England highlighted the inequity in access to services for patients:

*‘Some places are doing it and others aren’t. I don’t think it is working at the moment. Where’s the governance? Where’s the quality control? It’s just a free-for-all. It was a free-for-all before the pandemic and now it’s just even worse.’*

(P5, GP)

#### Infection prevention

Infection prevention concerns about transmission of COVID- 19 during spirometry procedures led to national guidance to support services to restart.^14^ Some described finding the guidance helpful, while others found it confusing or vague:

*‘So very much ARTP guidance, that risk minimisation in spirometry restart and ventilation etc. And also advice from PCRS* [Primary Care Respiratory Society] *was really helpful as well.’*

(P6, respiratory nurse)

*‘Not particularly* [helpful]*, because there was all talks about air changes. It was all a foreign language to many of us, certainly in primary care. And then once you even interpret what that means, how do you then implement that?’*

(P22, GP)

There was acknowledgement of the tension between being overly prescriptive and sufficiently flexible with guidance for restarting spirometry, to avoid being too restrictive for services:

*‘To be too prescriptive in the guidance that you’re giving then makes it very limiting for places … this is what we believe to be best practice, but we appreciate that you may need to tweak it slightly for your own areas.’*

(P23, physiologist)

Participants described wide variation in current spirometry provision in primary care, ranging from no restarting of services at all, to new models of delivering spirometry where measures were put in place to address infection control concerns:

*‘So essentially patients would drive into a marquee, complete the procedure and then drive off … we delivered it in a different way in an outdoor setting, which meant that we could actually deliver.’*

(P12, commissioner)

#### Competing priorities

The data highlighted a feeling of being overwhelmed in primary care at present, and the restart of spirometry being just one aspect of the many challenges faced:

*‘… but they* [primary care] *have multiple priorities … trying to recover a lot of long- term conditions, not just respiratory conditions, but cardiovascular, increased diagnoses of cancer as well as elective recovery. So it’s almost a perfect storm of issues meaning that it’s hard for them to focus on one specific area such as spirometry … I think it’s more a fact of where primary care is at the moment. It’s something that’s just too hard to do because it’s overwhelmed.’*

(P2, consultant)

Participants described the many competing priorities for services at this time and the pressure on workforce training and capacity:

*‘We have got some funding to retrain people but the difficulty is primary care is trying to restart everything at the same time and it’s competing priorities, it’s do they want to get vaccinations done, do they want to sort out diabetes, have they got somebody to do respiratory, so that’s the other problem.’*

(P3, respiratory nurse)

#### Impact on patients

All participants expressed concerns about the impact of the pandemic on spirometry services and consequences for patients and appropriate disease management:

*‘I think the biggest challenge is that we’re going to have a group of people with COPD, for example, who haven’t been diagnosed, and therefore not commenced on treatment or effective treatment, and we’re going to see them either presenting later in their disease or being diagnosed at the time of hospitalisation with an exacerbation.’*

(P4, consultant)

Some participants expressed concerns over not only regional inequity in spirometry, but also the inequity of all services for respiratory patients when compared with those with other diseases. Participants suggested this needs to be addressed to allow development of good spirometry pathways in future:

*‘It really worries me why it hasn’t been more of an urgent priority to sort it out really. I keep saying cardiologists wouldn’t put up with this. Imagine one of their key diagnostic tests was taken away from them.’*

(P9, consultant)

*‘The NHS would need to invest more in primary care respiratory services and that does mean investing in people, training people and making them enthusiastic about it.’*

(P17, respiratory nurse)

### Future delivery closer to patients’ homes by appropriately trained staff

All participants had clear ideas about what a good future pathway could look like, and this aligned to the delivery of spirometry in the right place, by people with the right skills. The right place was widely described as ‘close to home’:

*‘You want it to be accessible to patients, because obviously with things like inequalities people are at more risk of respiratory disease if they’re from lower socioeconomic groups. You don’t want them having to travel miles and miles to go to a hospital, so primary care is the right place.’*

(P8, GP)

There was a variety of innovative options put forward for new models of spirometry service delivery to make it close to home:

*‘The model that I want to see is a mobile diagnostic hub, whereby you have highly trained, ideally probably respiratory physiologists that do this day in, day out, on a bus. And probably a fleet of buses that has the IT system on board.’*

(P5, GP)

There were also examples of services that had recently been started to include PCN models, mobile spirometry, and diagnostic hubs:

*‘The patients now park up and walk up to the marquee and that was to overcome the ventilation issues around spirometry, you know the six air changes an hour, and it’s run by our confederation on behalf of our PCNs.’*

(P13, commissioner)

The right people to perform spirometry could be any variety of workforce if they were appropriately trained:

*‘The important thing is that whoever does it is trained and competent to do it … some really good healthcare assistants that work in extended roles under supervision, and that’s wholly appropriate and it’s more cost-effective and time-effective.’*

(P3, respiratory nurse)

### The potential for AI to have supportive roles in spirometry

Participants expressed their views on the roles of AI in spirometry quality assessment and interpretation, and how this had evolved as AI technology advances:

*‘Now that I’ve seen some of the newer artificial intelligence stuff I think it really does lend itself to supporting the community in being able to deliver better-quality assured spirometry in terms of the quality control.’*

(P23, physiologist)

Commissioners and clinicians from a variety of disciplines suggested that there may be a role for AI in the performance and interpretation of delivering quality-assured spirometry in primary care:

*‘I think we should embrace it. We’ve got a massive workforce shortage. If we can use some of this technology to do some of the things that we would historically have had to sit down and report, and it’s been shown to be beneficial, we don’t need to fear it.’*

(P9, consultant)

Key factors identified for whether it could have a supportive role were that it needs to be well validated, trusted by clinicians, cost- effective, to support workload, and to be an aid to existing pathways:

*‘I think it’s just got to have the buy-in of people using it, and there’s got to be the data and the evidence that it works … If you could have a really trustworthy artificial intelligence that has gone through all the right trials and you’ve got confidence in.’*

(P8, GP)

Possible barriers to acceptance included sceptical attitudes to technology and the terminology used to describe AI:

*‘I teach everybody never to trust a machine, so I think that I’m probably part of that issue.’*

(P16, respiratory nurse)

*‘I think it’s just people’s preconceptions about what AI is … people have different views, that there’s a physical robot walking around doing your test for you.’*

(P10, GP)

Those with expertise in interpreting spirometry, such as respiratory nurses with ARTP accreditation, respiratory physiologists, and respiratory consultants, appeared worried about clinicians who envision spirometry becoming deskilled with increased use of AI and less practice at interpreting results themselves. This highlights a misunderstanding of the role of AI as a supportive rather than replacement tool:

*‘I think you’ll always get a little bit of resentment from people if you tell them technology can do a better job.’*

(P14, respiratory nurse)

Concern was also expressed about the difference between technical interpretations of spirometry (for example, moderate obstruction) versus clinical diagnosis (for example, COPD) that requires additional information to spirometry results alone:

*‘You need to look at chest X-rays, bloods and clinical history, occupational history, everything right from the start, really. Which some people don’t understand and if this new algorithm says, “Oh, it’s fifty per cent chance or seventy per cent chance COPD”, they may just rely on that and not do anything else.’*

(P26, physiologist)

In contrast to the respiratory specialists, GPs suggested they would happily accept AI in helping them to interpret spirometry:

*‘I would not be surprised if AI were to interpret our own spirometry with more accuracy and less variation than we do …*

*So I think it would be a welcome addition providing there wasn’t a large cost barrier to this.’*

(P7, GP)

*‘It’s coming back to GPs who are generalists, they’re probably looking for a bit of support in making their diagnosis, and if the AI works and it is accurate then by all means, I think practices would enjoy that.’*

(P10, GP)

Many participants referred to the existing wide use of AI in other areas, such as electrocardiograms (ECGs), and how this has been fully integrated/accepted in routine care. Participants described how this can be used to build capacity for clinicians by screening out quickly those tests that are normal:

*‘ECGs are probably the one where it’s the most advanced and, you can put the sensitivity and specificity to certain levels so that when the AI says it’s normal, it’s definitely normal. And that’s incredibly helpful. Because you can then decrease the volume of interpretation as required by the physiologist or whoever’s doing it.’*

(P2, consultant)

Overall, there was a sense that there were supportive roles for AI to play in spirometry services, both in the conduct and the interpretation of spirometry:

*‘I think if there’s a tool that helps to support and improve the quality of the test and also the interpretation of it, then absolutely there’d be a key role for that.’*

(P4, consultant)

## DISCUSSION

### Summary

Interview data from key stakeholders involved in commissioning and delivering spirometry services outlined the challenges faced both historically and at present, with the restoration of services post-pandemic. The impact of the pandemic cannot be overestimated, not only on spirometry services but also on patient care, disease management, and primary care as a whole. AI may have roles to play in supporting spirometry pathways, particularly if it is able to improve quality assurance, reduce workload, and is trusted by clinicians.

The present study results highlight the current inequity in provision of spirometry services, and this must be urgently addressed to ensure that people with chronic lung disease receive correct diagnoses and management. The recent national changes in models of health care provide flexibility in the design and restoration of spirometry services; however, these data suggest that this may lead to further inequity, as those services with more resources and enthusiasm drive forward, leaving those without behind. These findings indicate possible opportunities to rethink spirometry pathways, workforce planning, and support from AI, to address both historical challenges and current difficulties in restarting spirometry post- pandemic. Despite some hesitancy around AI and clinicians deskilling, there is an overall sense that there is potential for AI to support clinicians in both quality assessment and interpretation. There is also a sense that clinicians and commissioners would embrace this tool, providing it has been validated, and has a positive impact on patient management, workload, and efficiency.

### Strengths and limitations

This work is current and undertaken at a time of extreme change for spirometry pathways, with an unprecedented halt in services and the introduction of new models of care in primary care. To the authors’ knowledge, this is the first qualitative work to describe clinician and commissioner perspectives on spirometry services in primary care relating to historical challenges and future opportunities. The authors interviewed key stakeholders from a variety of professional roles, with representation from different regions in England and from several national and regional specialist networks. Although participants alluded to previous knowledge of application of AI in various settings, they were not directly asked about previous understanding or experience of the application of AI in spirometry services. The aim was to engage with key stakeholders involved in spirometry; however, this comes at the expense of understanding the views of those working in primary care without a specific interest or enthusiasm for spirometry. The latter group may have expressed different views. The authors also acknowledge the value of patient perspectives as key stakeholders in spirometry services, which are lacking in this research, and plan to include this in the next stage of this wider programme of work.

### Comparison with existing literature

Many historical challenges faced by primary care in the provision of spirometry were highlighted in these qualitative data. The variation in quality of spirometry has been well documented,^4^ but this work highlights additional less well known issues related to funding, which have impacted how services were set up and delivered. One such issue is that spirometry has not been part of the General Medical Contract,^28^ and therefore payment is provided either by enhanced services contracts from clinical commissioning groups (CCGs) or via the Quality and Outcomes Framework (QOF) points linked to COPD diagnosis confirmed by quality-assured spirometry.^29^ No payment is received for test performance alone, and spirometry conducted at annual review receives no payment.

The move to integrated care systems rather than CCGs and the introduction of community diagnostic centres (CDCs) is intended to improve how spirometry is funded and to ensure that it is delivered by appropriately trained staff.^8^ However, these plans, laid out in the NHS Long Term Plan, have also been interrupted owing to the pandemic. The authors’ data confirm that the pre-pandemic model of spirometry was not working well. This accords with data from the Welsh national COPD audit, which demonstrated that only 19% of patients had gold standard post- bronchodilator spirometry recorded, and 25% of those diagnosed with COPD actually had spirometry incompatible with airway obstruction.^30^ From the authors’ data, commissioners and clinicians appear keen to use this opportunity for reorganisation of healthcare services for spirometry service redesign.

Many participants expressed that AI may have roles to play in providing spirometry in primary care by achieving quality assurance in both test performance and interpretation. However, it was evident that clear explanations of what AI software can do and how it works are necessary to improve trust, understanding, and buy-in to the process. A survey with healthcare staff to understand their knowledge and attitudes about AI revealed that, although 79% believed AI could support them in their daily work, a large proportion reported no previous use, and very low levels of understanding about the principles of AI.^31^

There are many successful applications of AI in health care, particularly in electrocardiography, radiology, and symptom recognition tools where it has been shown to improve accuracy of screening and reduce workload.^32^^,^^33^ The ArtiQ.Spiro software can indicate errors in the performance of spirometry testing, the overall quality of the trace, aid in technical interpretation by performing pattern recognition, and indicate a likelihood of disease along with prompts for next steps.^34^

Tension appears to exist between possible over-reliance on AI resulting in reduced clinical judgement or deskilling, and potential benefits of AI aiding clinicians in interpretation and less time spent on more straightforward tasks (for example, sifting out all normal spirometry traces) thereby allowing clinicians to focus on more complex patient management. Those participants with more general roles (GPs) were less worried about the possibility of deskilling, and were more likely to see the value for them in using AI as an aid. Other research exploring patient perspectives on AI in healthcare has revealed perceived benefits such as greater accuracy, reduced workforce burden, and equality of healthcare decision making, while acknowledging concerns about data cybersecurity and limitations of technology.^35^

### Implications for research and practice

Current spirometry provision appears to be characterised by extreme variations and fragmentation in services in different areas of England, ranging from no spirometry service at all and long waiting lists, to initiatives involving drive-through spirometry and mobile hubs. Restoration of services will have to address a large backlog of patients, estimated to be 200– 250 patients per 500 000.^14^ In addition to patients awaiting diagnostic spirometry, there are those patients who require testing as part of their annual review.^2^ It remains unclear how this will fit in with PCN and CDC spirometry delivery, or if it will widen the gap in existing inequity of care for respiratory patients.^36^

Challenges described in restarting spirometry included workforce capacity, ongoing funding issues, and practical considerations, such as how to safely deliver the test while minimising infection risks. The mixed response from participants to national guidance regarding restarting and infection prevention measures^14^ may reflect the different levels of expertise and resources in different locations.

It was clear that spirometry should be delivered close to home for patients by appropriately trained staff. Regardless of the details of a service model, all participants expressed the importance of spirometry being accessible for patients without travelling far, to address current inequity in care for patients.

Any clinician could be appropriately trained to regularly perform spirometry; familiarity and frequency of testing and interpretation have been demonstrated as beneficial in a previous study.^37^ Work may be needed to increase support in practice for the ARTP accreditation process. Good- quality spirometry test performance is the key to obtaining meaningful results to aid diagnosis, therefore, even with AI reporting, there is still a need to ensure that practitioners are skilled and spirometry equipment is maintained, calibrated, and cleaned appropriately. AI may be well placed to support practitioners to identify errors in test performance in real time and encourage improved patient technique.

Research is needed to explore whether a spirometry provision in primary care incorporating AI increases capacity among clinicians in a cost-effective manner; for example, by quickly reporting all normal test results and prompting where further investigation is required. Work is also needed to establish whether using AI to aid interpretation leads to earlier and more accurate diagnoses, which is a huge priority for improving patient quality of life, reducing avoidable healthcare visits and inappropriate prescribing, and is in line with the national ambition to reduce the environmental impact of respiratory health care.^38^ Future research will also need to explore the new models of care proposed in primary care to evaluate the effectiveness of service delivery, quality of spirometry in diagnostic hubs, accuracy of interpretation, and also perceived positives and negatives from a patient and clinician perspective.

## Data

The data sets generated and analysed during the current study are available from the corresponding author on reasonable request.
